# Characterisation of the Myocardial Mitochondria Structural and Functional Phenotype in a Murine Model of Diabetic Cardiomyopathy

**DOI:** 10.3389/fphys.2021.672252

**Published:** 2021-09-01

**Authors:** Alex M. Parker, Mitchel Tate, Darnel Prakoso, Minh Deo, Andrew M. Willis, David M. Nash, Daniel G. Donner, Simon Crawford, Helen Kiriazis, Cesare Granata, Melinda T. Coughlan, Miles J. De Blasio, Rebecca H. Ritchie

**Affiliations:** ^1^Heart Failure Pharmacology, Drug Discovery Biology, Monash Institute of Pharmaceutical Sciences, Monash University, Melbourne, VIC, Australia; ^2^Baker Heart & Diabetes Institute, Melbourne, VIC, Australia; ^3^Department of Pharmacology and Therapeutics, The University of Melbourne, Melbourne, VIC, Australia; ^4^Baker Department of Cardiometabolic Health, The University of Melbourne, Melbourne, VIC, Australia; ^5^Ramaciotti Centre for Cryo-Electron Microscopy, Monash University, Melbourne, VIC, Australia; ^6^Department of Diabetes, Monash University, Melbourne, VIC, Australia; ^7^Institute for Health and Sport, Victoria University, Melbourne, VIC, Australia; ^8^Department of Pharmacology, Monash University, Melbourne, VIC, Australia

**Keywords:** diabetes, heart, experimental – animal models, mitochondria, diabetic cardiomyopathy, mitochondrial function

## Abstract

People affected by diabetes are at an increased risk of developing heart failure than their non-diabetic counterparts, attributed in part to a distinct cardiac pathology termed diabetic cardiomyopathy. Mitochondrial dysfunction and excess reactive oxygen species (ROS) have been implicated in a range of diabetic complications and are a common feature of the diabetic heart. In this study, we sought to characterise impairments in mitochondrial structure and function in a recently described experimental mouse model of diabetic cardiomyopathy. Diabetes was induced in 6-week-old male FVB/N mice by the combination of three consecutive-daily injections of low-dose streptozotocin (STZ, each 55 mg/kg i.p.) and high-fat diet (42% fat from lipids) for 26 weeks. At study end, diabetic mice exhibited elevated blood glucose levels and impaired glucose tolerance, together with increases in both body weight gain and fat mass, replicating several aspects of human type 2 diabetes. The myocardial phenotype of diabetic mice included increased myocardial fibrosis and left ventricular (LV) diastolic dysfunction. Elevated LV superoxide levels were also evident. Diabetic mice exhibited a spectrum of LV mitochondrial changes, including decreased mitochondria area, increased levels of mitochondrial complex-III and complex-V protein abundance, and reduced complex-II oxygen consumption. In conclusion, these data suggest that the low-dose STZ-high fat experimental model replicates some of the mitochondrial changes seen in diabetes, and as such, this model may be useful to study treatments that target the mitochondria in diabetes.

## Introduction

The global prevalence of diabetes mellitus in adults has increased from an estimated 108 million in 1980, to 422 million by 2014, and is projected to reach 693 million by 2045 (Cho et al., 2018). Type 2 diabetes is the predominant type, accounting for approximately 90% of cases in adults (Zhou et al., 2016). Heart failure incidence is roughly 3-times greater in patients with concomitant diabetes, compared to patients without diabetes (Nichols et al., 2004). Furthermore, diabetes patients have considerably worse clinical outcomes associated with heart failure, in contrast to those without diabetes (Jia et al., 2018). This is in part explained by a distinct form of heart failure that can manifest in diabetes patients, termed diabetic cardiomyopathy. Diabetic cardiomyopathy is typified by maladaptive changes in cardiac structure and function that occur independently of other cardiovascular risk factors, including hypertension, coronary artery disease, and atherosclerosis (Rubler et al., 1972; Ritchie and Abel, 2020). Despite our progress in understanding this disease process, there are currently no effective treatments that specifically target the underlying pathogenesis contributing to diabetic cardiomyopathy (Marwick et al., 2018).

Excess levels of reactive oxygen species (ROS) are a common feature of the diabetic heart, and are thought to accelerate the progression of diabetic cardiomyopathy (Teshima et al., 2014; Ritchie and Abel, 2020). In diabetes, as endogenous antioxidants become overwhelmed, there is typically an imbalance between ROS generation and ROS removal, leading to oxidative stress in the heart (Liu et al., 2014; Xu et al., 2017). Importantly, mitochondria are a major site of ROS generation in the heart, due to the high energy demand and oxygen consumption (Kaludercic and Di Lisa, 2020). This is further exacerbated by metabolic inflexibility in diabetes, where there is abnormal myocardial substrate utilisation (Amaral and Okonko, 2015). Ultimately, these conditions trigger mitochondrial dysfunction, leading to increased electron leakage from the mitochondrial respiratory chain, potentiating excessive levels of ROS (Fillmore et al., 2014).

Conventional, widely-used experimental models of type 2 diabetes, such as the *db/db* mouse and *ob/ob* mouse, display increased fatty acid oxidation in the cardiac mitochondria, leading to mitochondrial dysfunction and oxidative stress (Mazumder et al., 2004; Boudina et al., 2007). However, these models possess several confounding factors, including impaired leptin signalling and supra-pathological obesity, that represent important limitations (Barouch et al., 2003; Burke et al., 2017). In a previous study from our laboratory, we described an alternative model of diabetic cardiomyopathy, that combined low-dose streptozotocin (STZ) with high-fat diet (Tate et al., 2019). Importantly, this experimental model has a milder systemic phenotype than the genetic models described above, whilst replicating several of the key clinical features of diabetic cardiomyopathy, including robust diastolic dysfunction and characteristic changes in myocardial structure in the context of elevated body weight and impaired glucose tolerance (Tate et al., 2019). Whilst this earlier study reported changes in the expression of genes associated with left ventricular (LV) mitochondrial function (Tate et al., 2019), the direct impact on mitochondrial morphology was beyond its scope. Therefore, this study sought to investigate the structure and function of cardiac mitochondria in this murine model of diabetic cardiomyopathy.

## Materials and Methods

### Animals

The use of mice for this study was approved by the Alfred Medical Research and Education Precinct (AMREP) Animal Ethics Committee. All research activities involving animals were in accordance with the guidelines provided by the National Health and Medical Research Council of Australia for animal experimentation. Mice were sourced, housed, and maintained in the AMREP Animal Centre under a 12-h light/dark cycle at 22°C ± 1°C with access to food and water.

### Experimental Design and Assessment of Systemic Phenotype

The primary aim of this study was to characterise the structure and function of cardiac mitochondria in a model of diabetic cardiomyopathy that combines low-dose STZ and high-fat diet (and which replicates many aspects of human type 2 diabetes as a result; Tate et al., 2019). We have included flow charts for reporting animal use in all experiments (Supplementary Figure 1). FVB/N male mice (6-week-old) were randomly allocated to the non-diabetic or diabetes group. To induce diabetes, mice received three consecutive-daily intraperitoneal (i.p.) injections of STZ (55 mg/kg/day, in 0.1 mol/L citric acid vehicle dissolved in saline). STZ-administered mice were then placed on a high-fat diet (42% energy from lipids; SF04-001, Speciality Feeds) for the remainder of the study (to induce diabetes, impair glucose tolerance and elevate body weight). Control mice received citrate vehicle followed by a standard laboratory chow diet for the study duration. Fortnightly blood glucose measurements using a glucometer (Accu-Chek, Roche) were carried out to assess the presence of diabetes via the saphenous vein. Diabetic mice received daily monitoring and husbandry. Toward study end, mice were individually housed in Promethion metabolic cages for 24 h (Sable Systems International). Individual cages were thermally controlled and contained a ceiling-mounted food hopper and water bottle. Cages contained a lid-mounted house that records body mass. A running wheel was available for the duration of the experiment (Soto et al., 2019). Whole body composition was analysed using an Echo-MRI^TM^ 4-in-1 700 Analyzer one week before tissue collection, as previously described (Tate et al., 2019). Intraperitoneal glucose tolerance tests (IPGTT) were performed one week prior to tissue collection. Mice were fasted for 5 h before recording baseline blood glucose levels. After mice received a glucose challenge (25% i.p.), blood glucose levels were measured using a glucometer (Accu-Chek, Roche) at 15, 30, 45, 60, 90, and 120 min after collecting a drop of blood from the saphenous vein (Tate et al., 2019). Glucose clearance rate was later determined by calculating area-under-the-curve (AUC) using the baseline blood glucose concentration for each experimental group. A minimum 24-h rest period was included between experiments. At study endpoint, glycated haemoglobin (HbA1c) analysis was performed using the Cobas-b-101 POC system (Roche). Prior to tissue collection, mice were anaesthetised using ketamine/xylazine (85/8.5 mg/kg i.p.) followed by exsanguination via rapid removal of the heart. All mice were sacrificed for tissue collection at approximately the same time of each day (always between 9am and 12pm) to minimise any influence due to circadian effect. LV was dissected for immediate superoxide measurements, histological processing, morphological and functional assessment of mitochondria or snap-frozen in liquid nitrogen and stored at −80°C for subsequent biochemical analysis.

### Assessment of Cardiac Phenotype: Fibrosis, ROS Detection, and LV Function

#### Myocardial Histology

LV tissues were placed in 10% neutral buffered formalin for 24 h before being embedded in paraffin. Paraffin-embedded LV tissues were cut (4 μm thick) and stained by the Monash University Histology Platform. Images were collected using an Olympus BX60 microscope. Picrosirius red stain was used to assess the level of LV interstitial collagen (polarised microscopy was used to specifically identify type I and III collagen). Scanned images were blinded then quantified using ImageJ software (picrosirius red at ×200 magnification, 10–32 images per sample; Tate et al., 2019).

#### Myocardial ROS Detection

LV superoxide levels were detected via lucigenin-enhanced chemiluminescence. During tissue collection, fresh LV sections (4 mm^3^ × 1 mm^3^ per sample) were placed into individual wells of an opaque 96-well optiplate (Perkin Elmer) containing 100 μL of Krebs-HEPES buffer. β-NADPH was added to three of the four tissue-containing wells, assigning one as the non-substrate control. After a 1-h incubation at 37°C, lucigenin (5 μM) was added to every well, before being placed into an EnSpire Plate reader (Perkin Elmer) for superoxide detection via chemiluminescence (Tate et al., 2019). An Amplex Red assay kit (Invitrogen) was used to assess LV hydrogen peroxide content, as per manufacturer’s instructions. Hydrogen peroxide standards and LV protein samples were added to a black 96-well plate (Sigma-Aldrich). Standards and samples were then incubated for 30 min with Amplex Red/horseradish peroxidase (0.1 mM/0.2 U/ml, respectively) in the dark, prior to hydrogen peroxide detection using a fluorescence CLARIOstar plate reader (530 nm/590 nm excitation/emission).

#### Myocardial Function

One week prior to tissue collection, echocardiography was carried out in anaesthetised mice (ketamine/xylazine/atropine; 80/8/0.96 mg/kg i.p.) using the Vevo 2100 ultrasound machine (VisualSonics). Echocardiography was performed (technicians were blinded to treatment group) and validated by the Baker Institute Preclinical Cardiology Platform (Donner et al., 2018). M-mode function was used to assess LV chamber dimensions and fractional shortening. The B-mode function was used to measure LV volume and area during the systolic and diastolic phase to quantify ejection fraction, and cardiac output. The transmitral Doppler flow function was used to assess LV filling (early [E] and late [A] filling), E/A ratio and deceleration time. Tissue Doppler echocardiography technique was used to evaluate tissue velocity (early [e’] and late [a’] filling) and the e’/a’ ratio.

#### Morphological Analysis of Mitochondria

At the time of tissue collection, fresh tissues were dissected (4 pieces each approximately 1 mm^3^) from the apex of LV and placed in microfuge tubes (Eppendorf) containing 1 mL of fixative (2% paraformaldehyde, 2.5 glutaraldehyde in 0.1 M sodium-cacodylate buffer), before being processed by the Monash Ramaciotti Centre for Cryo-Electron Microscopy. Imaging was carried out by the Monash Ramaciotti Centre for Cryo-Electron Microscopy. Only one of the four pieces was utilised for imaging by electron microscopy. From this we obtained 10 images per mouse heart sample and analysed them using ImageJ software with the investigator blinded to the experimental groups. Total number of mitochondria or lipid droplets per image were counted and averaged per sample. The mitochondrial width (Feret diameter), area, length and aspect ratio, and lipid droplet count, were analysed.

#### Abundance of Mitochondrial Respiratory Chain Proteins

The abundance of mitochondrial respiratory chain proteins was determined via Western blot analysis of key subunits in each of complexes I-V. Protein was homogenised in ice-cold RIPA buffer using the Tissue Lyser II machine (Qiagen) at 30 Hz for 1 min. 30 μg of protein was loaded into 4–15% Tris-glycine gradient gel (Cat. no. 4561026, Bio Rad; 4–15% Mini-PROTEAN^®^ TGX Protein Gels) and separated by SDS-PAGE gel electrophoresis. To assess relative protein abundance of mitochondrial complexes I-V, the membrane was incubated overnight with the Total OXPHOS Rodent WB Antibody Cocktail (Cat. no. ab110413, Abcam; 1:1000 dilution) or Anti-Calnexin C-Terminal Rabbit Polyclonal Antibody housekeeper (Cat. no. 208880, Abcam; 1:1000) at 4°C. A 60-min incubation was then carried out using a polyclonal rabbit anti-mouse immunoglobulins/horseradish peroxidase secondary antibody (1:3000 dilution, Dako), prior to membrane imaging using the ChemiDoc imaging system (Bio-Rad), and quantification using Image Lab software.

#### Assessment of Myocardial Mitochondrial Respiration

At study end, freshly dissected LV tissue was permeabilised in saponin (50 μg/mL)/biopsy preservation solution (BIOPS: 10 mM Ca-EGTA buffer, 0.1 μM free calcium, 20 mM imidazole, 20 mM taurine, 50 mM K-MES, 0.5 mM DTT, 6.56 mM MgCl_2_, 5.77 mM adenosine triphosphate (ATP), 15 mM phosphocreatine, pH 7.1) for 20 min at 4°C with gentle rocking, as previously described (Horscroft et al., 2015). Tissues were washed three times (5-min intervals) with mitochondrial respiratory medium (MiR05: 0.5 mM EGTA, 3 mM MgCl_2_.6H_2_O, 60 mM K-lactobionate, 20 mM taurine, 10 mM KH_2_PO_4_, 20 mM HEPES, 110 mM sucrose, 1 g/L, defatted BSA, pH 7.4; (Horscroft et al., 2015). Samples (1–2 mg) were then placed in the Oxygraph-O2k (Oroboros Instruments, Innsbruck, Austria) chambers containing 2 mL MiR05 at 37°C. A substrate-uncoupler-inhibitor titration (SUIT) protocol (Pesta and Gnaiger, 2012) was used as follows: 5 mM Glutamate and 10 mM malate were added to assess leak respiration (L) in the absence of adenylates and limitation of flux by electron input through complex I (CI_*LEAK*_); 1 mM adenosine diphosphate was added to asses OXPHOS capacity [P] with limitation of flux by electron input through CI (CI_*OXPHOS*_); complex I was then inhibited by addition of 0.5 μM rotenone, and 10 mM succinate were subsequently added to asses [P] with limitation of flux by electron input through CII (CII_*OXPHOS*_); 10 μM cytochrome *c* was added to test for outer mitochondrial membrane integrity (not shown in representative trace), before final addition of 5 μM antimycin A to determine the residual non-mitochondrial oxygen consumption [ROX]. Oxygen concentration in the chambers was maintained between 250 and 400 μM. Data of mitochondrial respiration were normalised by wet tissue mass and are presented as [pmol O2/s/mg wet weight].

#### Gene Expression Analysis

RNA extraction was performed using the commercially-available GenElute^TM^ Mammalian Total RNA Miniprep Kit (Sigma-Aldrich) and reversed-transcribed using the high-capacity cDNA reverse-transcription kit (Thermo Fisher Scientific), both as per the manufacturer’s instructions. LV gene expression was carried out using real-time PCR with SYBR green chemistry (ThermoFisher). Primers were generated using Primer3 Plus from mouse sequences in PubMed gene database (Supplementary Table 1). Relative gene expression was detected and quantified via QuantStudio7 Flex system (Applied Biosystems), using the comparative delta-delta cycle threshold (ΔΔCT) method to determine fold-change relative to non-diabetic mice.

#### Statistical Analysis

Data analysis was performed using GraphPad Prism 9.0.0 statistical software. Comparisons between treatment groups were analysed using unpaired *t*-test. *P* < 0.05 was considered statistically significant.

## Results

### Confirmation of Systemic Phenotype in Diabetic Mice

**FIGURE 1:**
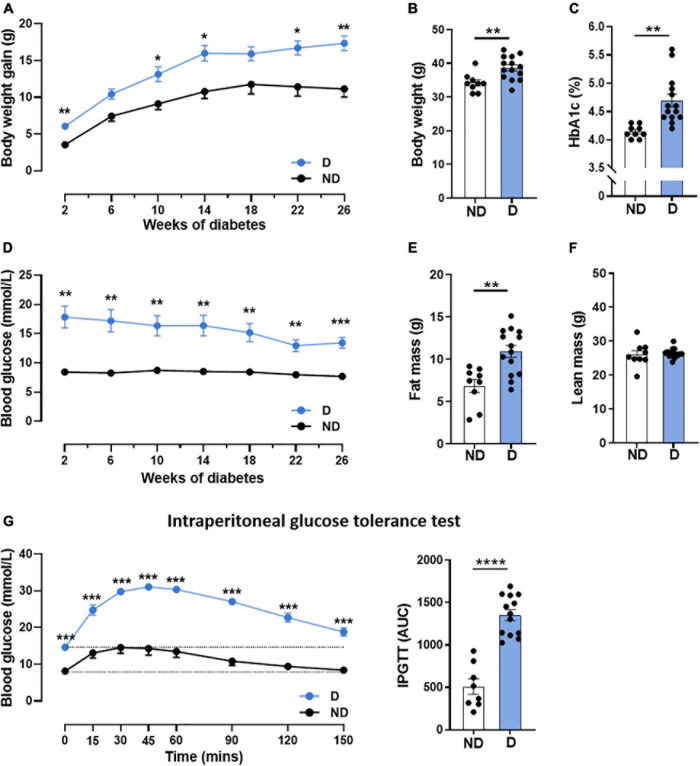
Characterisation of systemic phenotype in diabetic mice. **(A)** Body weight gain over time, **(B)** end-point body weight, **(C)** glycated haemoglobin (HbA1c), **(D)** blood glucose over time, **(E)** fat mass, **(F)** lean mass, **(G)** intraperitoneal glucose tolerance test (IPGTT); dotted line indicates basal glucose for each group where AUC is calculated. Data represented as mean ± SEM. *n* = 8–14 per group (note individual data points). Data analysis used unpaired *t*-test. *P < 0.05, **P < 0.01, ***P < 0.001, ****P < 0.0001. ND, non-diabetic; D, diabetes; AUC, area under curve.

Patients with diabetes commonly exhibit an increase in blood glucose with altered glucose handling and weight gain (Ritchie and Abel, 2020). Hence, we first confirmed that diabetic mice receiving high-fat diet in combination with STZ in this study exhibited significantly higher weight gain throughout the 26 weeks of diabetes (Figure 1A), leading to an increase in body weight at study end (Figure 1B). No differences were seen in tibial length, a marker of animal size, between diabetic mice and non-diabetic mice (Supplementary Table 2). At study end, diabetic mice had elevated glycated haemoglobin (HbA1c) levels, a measure of long-term blood glucose levels (Figure 1C). Diabetic mice also exhibited a significant increase in blood glucose levels by the 2-week timepoint, and this remained elevated for the duration of the study (Figure 1D). Whole body composition analysis in conscious mice was assessed using Echo-MRI; this revealed a significantly increased fat mass in diabetic mice, compared to non-diabetic control mice (Figure 1E), however, there was no difference in lean mass between groups (Figure 1F). Glucose tolerance tests are a routine clinical method to assess glucose handling (Marwick et al., 2018). Glucose tolerance was significantly impaired in diabetic mice over the course of 150 min, as demonstrated by the larger area-under-the-curve (Figure 1G). Diabetic mice also exhibited larger liver and spleen weights (Supplementary Table 2) compared to their non-diabetic counterparts. The weights of individual fat pads, including peri-renal and inguinal fat, were also significantly elevated in diabetic mice (Supplementary Table 2).

### Confirmation of Cardiac Phenotype in Diabetic Mice

**FIGURE 2:**
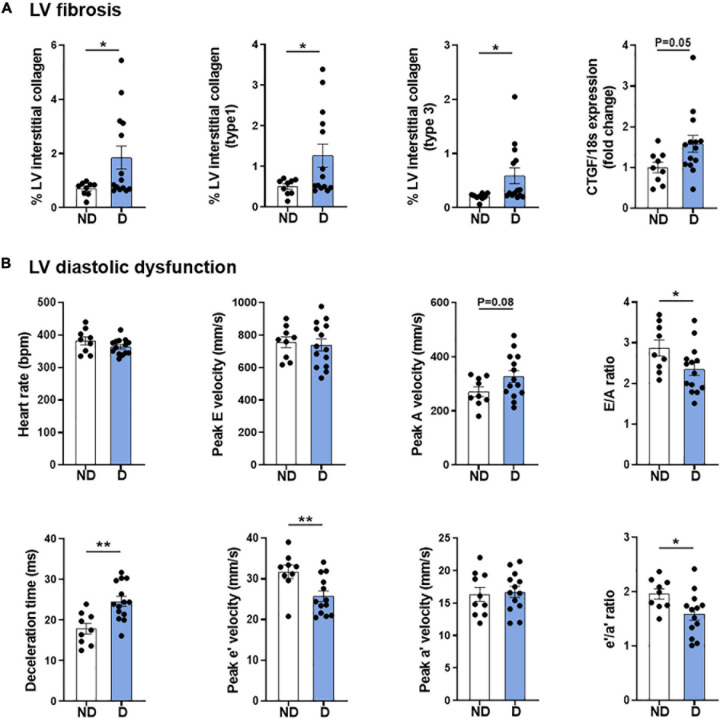
Cardiac phenotype of diabetic mice. **(A)** LV fibrosis: Total interstitial collagen, type 1 collagen and type 3 collagen analysis using polarised light in picrosirius-red stained LV sections, and gene expression of fibrosis marker connective tissue growth factor (CTGF). **(B)** LV diastolic function: Heart rate, peak E wave velocity, peak A wave velocity, E/A ratio, deceleration time, peak e’ wave velocity, peak a’ wave velocity, and e’/a’ ratio. Data represented as mean ± SEM. *n* = 9–14 per group (note individual data points). Data analysis used unpaired *t*-test. *P < 0.05, **P < 0.01. ND, non-diabetic; D, diabetes; H_2_O_2_, hydrogen peroxide; LV, left ventricle; NOX, NADPH oxidase; ROS, reactive oxygen species.

As described previously, this model combining low-dose STZ superimposed on high-fat diet mimics several features of diabetic cardiomyopathy, including LV fibrosis and ROS generation, and most importantly, robust LV diastolic dysfunction at 26 weeks (Figure 2; Tate et al., 2019). Corroborating observations from our previous publication using a different cohort of mice (Tate et al., 2019), whole heart, LV, right ventricle, and atria weights were unchanged with diabetes (Supplementary Table 2). These observations corresponded with characteristic cardiac structural changes typically observed in diabetic cardiomyopathy, including an increase in myocardial fibrosis, as highlighted by an increase in LV interstitial collagen deposition (Figure 2A), and an increase in the mRNA expression of pro-fibrotic growth factor, connective tissue growth factor (CTGF; Figure 2A). As mentioned, diastolic dysfunction was present in diabetic mice as highlighted by several markers, including E/A ratio, deceleration time and e’/a’ ratio (Figure 2B). Importantly, heart rate was the same in both groups (Figure 2B).

### Impact of Diabetes on Systemic Energy Expenditure

**FIGURE 3:**
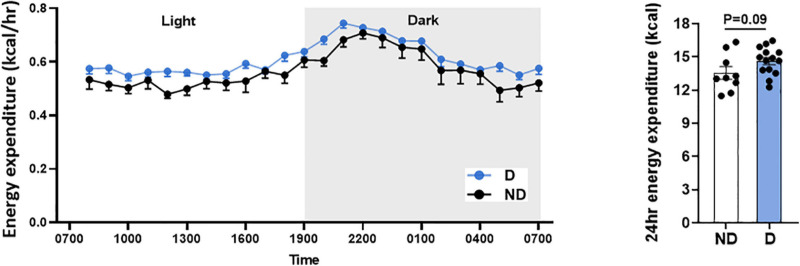
Energy expenditure over 24 h in diabetic mice. Data represented as mean ± SEM. *n* = 9–14 per group (note individual data points). Data analysis used unpaired *t*-test. ND, non-diabetic; D, diabetes.

Type 2 diabetes patients commonly have higher energy expenditure (Bitz et al., 2004). In this study, there was a tendency toward higher energy expenditure over 24 h in diabetic mice, however, this was not statistically different (Figure 3).

### Impact of Diabetes on LV ROS Production

**FIGURE 4:**
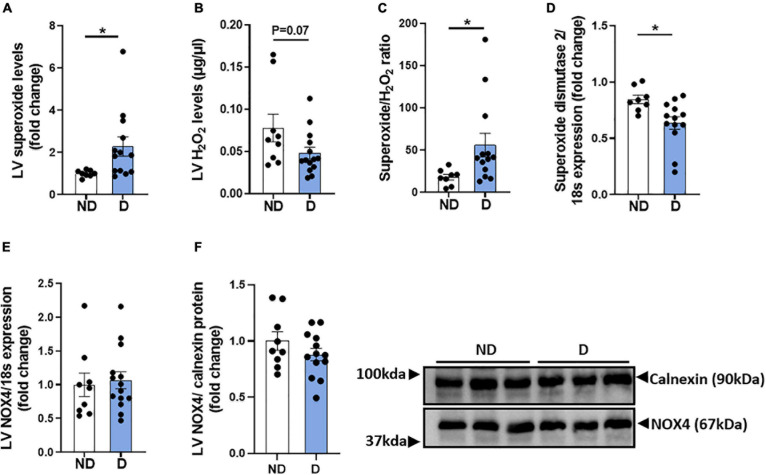
LV ROS production in diabetic mice. **(A)** LV Superoxide generation, **(B)** LV H_2_O_2_ generation, **(C)** LV superoxide/H_2_O_2_ ratio. Markers of LV ROS generation; gene expression of **(D)** superoxide dismutase-2 and **(E)** NADPH oxidase-4 (NOX4), and **(F)** protein abundance of NOX4. Data represented as mean ± SEM. *n* = 8–14 per group (note individual data points). Data analysis used unpaired *t*-test. *P < 0.05. ND, non-diabetic; D, diabetes.

Diabetes-induced ROS generation and subsequent oxidative stress are contributing factors in the development and progression of diabetic cardiomyopathy (Jia et al., 2018). LV Superoxide levels were elevated in diabetic mice in this experimental model of diabetic cardiomyopathy (Figure 4A). There was also a trend toward a reduction in hydrogen peroxide levels in diabetic mice, compared to non-diabetic mice (Figure 4B). Accordingly, diabetic mice exhibited a significant increase in the LV superoxide/hydrogen peroxide ratio (Figure 4C). LV mRNA expression levels of superoxide dismutase-2 was significantly lower in diabetic mice, compared to non-diabetic mice (Figure 4D). No differences were observed in LV mRNA expression or protein abundance of the NADPH-oxidase subunit NOX4 (Figures 4E,F).

### Abundance of Mitochondrial Respiratory Chain Proteins in Diabetic Mice

**FIGURE 5:**
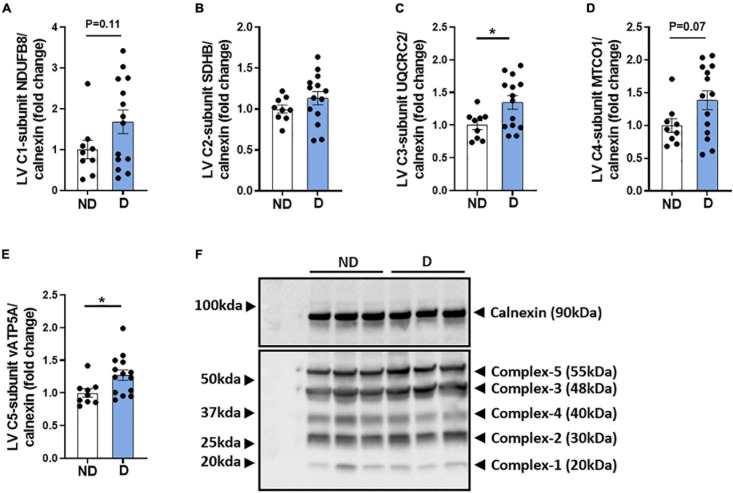
Protein abundance of mitochondria complexes in diabetic mice. Western blot quantification of **(A)** LV complex-I-subunit NDUFB8/calnexin, **(B)** LV complex-II-subunit SDHB/calnexin, **(C)** LV complex-III-subunit UQCRC2/calnexin, **(D)** LV complex-IV -subunit MTCO1/calnexin, **(E)** LV complex-V -subunit vATP5A/calnexin. **(F)** Representative blot images of mitochondria complexes. Data represented as mean ± SEM. *n* = 9–14 per group (note individual data points). Data analysis used unpaired *t*-test. *P < 0.05. ND, non-diabetic; D, diabetes; LV, left ventricle.

The mitochondria respiratory chain is a major contributor to excess ROS levels and the subsequent oxidative damage that is characteristic of the diabetic heart (Ding et al., 2019; Kaludercic and Di Lisa, 2020). There was a tendency toward an increase in complex-I protein abundance in diabetic mice (Figure 5A). No difference was observed in complex-II abundance between groups (Figure 5B). Diabetic mice exhibited a significant increase in complex-III protein abundance (Figure 5C), a tendency for elevated complex-IV protein abundance (Figure 5D), and a significantly elevated complex-V protein abundance (Figure 5E). Representative images of individual mitochondrial protein complex abundance in diabetic and non-diabetic mice are shown (Figure 5F).

### Characterisation of LV Mitochondrial Morphology in Diabetic Mice

**FIGURE 6:**
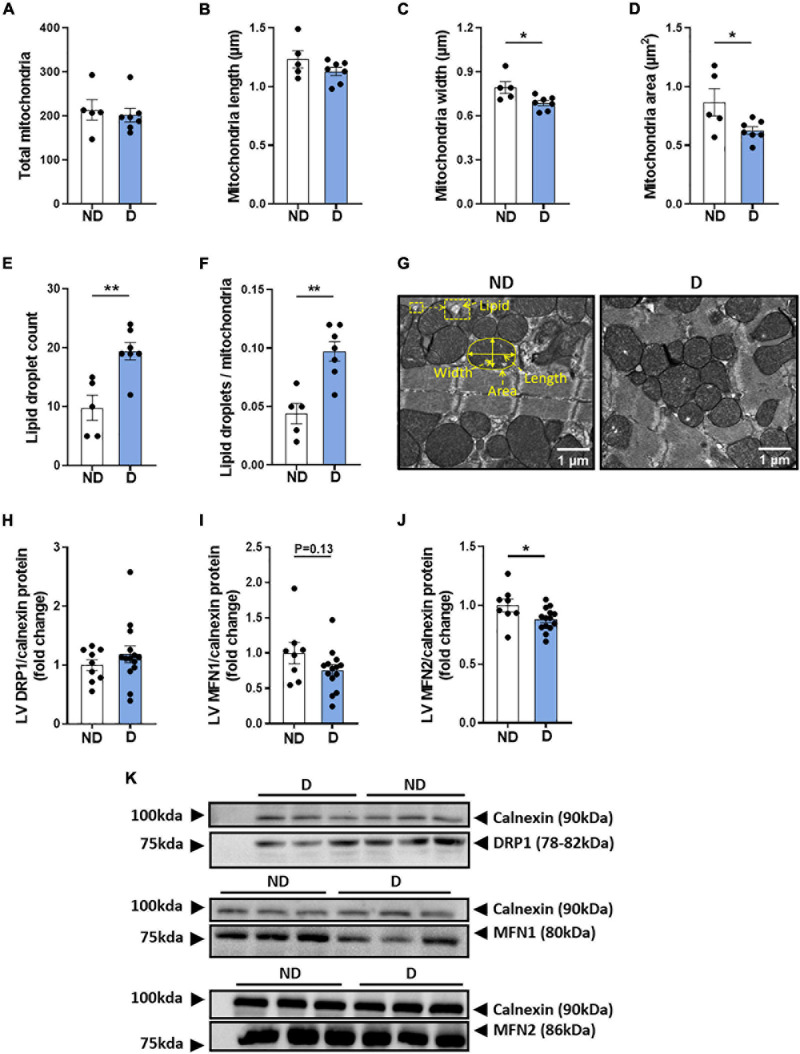
LV mitochondria morphology in diabetic mice. Electron microscopy quantification of **(A)** total number of mitochondria, **(B)** mitochondria length, **(C)** mitochondria width, **(D)** mitochondria area, **(E)** total lipid droplet count, **(F)** lipid droplets/mitochondria ratio, **(G)** representative electron microscopy images of mitochondria structure (an enlarged image is available to view in Supplementary Figure 2). Regulators of mitochondrial morphology: **(H)** dynamin-related protein 1 (DRP1) and **(I)** Mitofusin 1 (MFN1), **(J)** Mitofusin 2 (MFN2), with **(K)** representative western blot images; Calnexin housekeeper are carried out separately for each protein of interest. Data represented as mean ± SEM. *n* = 5–14 per group (note individual data points). Data analysis used unpaired *t*-test. *P < 0.05, **P < 0.01. ND, non-diabetic; D, diabetes; LV, left ventricle.

Changes in mitochondrial ultrastructure have previously been observed in the diabetic heart, in both the clinical and experimental setting (Dabkowski et al., 2010; Montaigne et al., 2014; Daghistani et al., 2019). In the current study, no differences were observed in the total number of mitochondria, or in mitochondrial length between experimental groups (Figures 6A,B). Interestingly, diabetic mice showed a significant decrease in mitochondria width and area (Figures 6C,D). The mitochondrial aspect ratio was not altered by diabetes (ND: 0.646 ± 0.009; D: 0.616 ± 0.017, NS). The total number of lipid droplets was elevated in diabetic mice (Figure 6E). Accordingly, the lipid droplet number to mitochondria ratio was significantly higher in diabetic mice (Figure 6F). Representative electron microscopy images of cardiac mitochondria are displayed (Figure 6G). Western blot analysis showed no differences in the protein expression of LV dynamin-related protein 1 (DRP1) (Figure 6H), however, there was a tendency for diabetic mice to exhibit lower protein expression of LV Mitofusin 1 (MFN1) (Figure 6I), and a significant reduction in LV Mitofusin 2 (MFN2) (Figure 6J). Representative western blot images are presented (Figure 6K).

### Characterisation of Cardiac Mitochondrial Respiratory Function in Diabetic Mice

**FIGURE 7:**
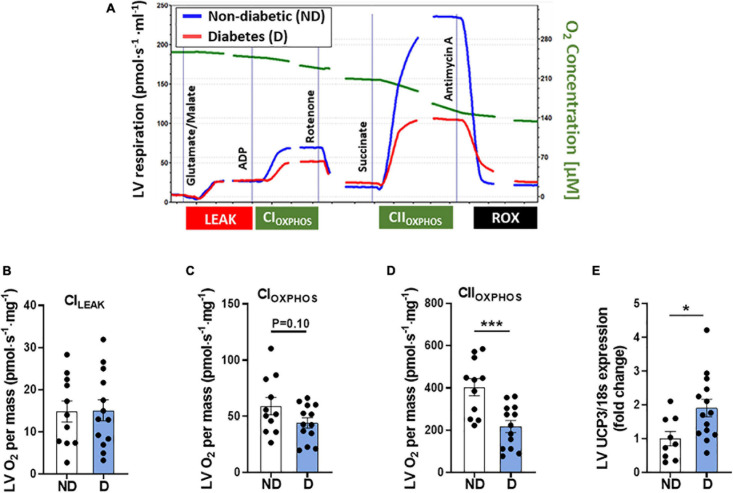
LV mitochondria function in diabetic mice**. (A)** Representative mitochondrial oxygen consumption trace in diabetic mice (red line) and non-diabetic mice (blue line). Image has been cropped where lines are broken. Cytochrome C addition not shown. Oroboros high-resolution respirometry analysis of: **(B)** Complex-I^*LEAK*^ respiration, **(C)** Complex-I oxygen consumption, **(D)** Complex-II oxygen consumption. LV gene expression of **(E)** mitochondrial uncoupling protein-3 (UCP3). Data represented as mean ± SEM. *n* = 9–14 per group (note individual data points). Data analysis was carried out using unpaired *t*-test. *P < 0.05, ***P < 0.001. ND, non-diabetic; D, diabetes; LV, left ventricle.

Changes in cardiac mitochondrial electron transport chain activity and mitochondrial respiration have previously been observed in patients with diabetes (Montaigne et al., 2014). In this study high resolution respirometry was carried out using the Oroboros Instruments Oxygraph-O2k to assess cardiac mitochondrial function. Figure 7A displays a representative trace of oxygen consumption over time in non-diabetic and diabetic mice. No differences were seen in [CI]_*LEAK*_ between groups (Figure 7B), however, there was a tendency toward reduced CI-linked mitochondrial respiration [CI]_*OXPHOS*_ in diabetic mice (Figure 7C). Diabetic mice also displayed a significant decrease in CII-linked mitochondrial respiration (Figure 7D). Gene expression analysis of LV tissue revealed a significant increase in mitochondrial uncoupled protein-3 in diabetic mice (UCP3; Figure 7E), compared to non-diabetic mice.

## Discussion

This study builds upon previous reports from our laboratory describing an experimental model of diabetic cardiomyopathy that recapitulates several of the clinical features of human disease, including robust diastolic dysfunction and myocardial structure changes. The model combines a low-dose STZ approach with high-fat diet (Tate et al., 2019). Mitochondrial dysfunction and an overproduction of ROS are common features of the diabetic heart, as well as major drivers of cardiac remodelling (Gollmer et al., 2020). In our previous study we noted changes in the expression of genes associated with LV mitochondrial respiratory function, providing a rationale to study mitochondria structure and function in more detail in this model (Tate et al., 2019). Mitochondrial dysfunction and oxidative stress are present in commonly-utilised experimental models of diabetes, including the *db/db* and *ob/ob* spontaneous genetic models (Mazumder et al., 2004; Boudina et al., 2007). However, these models have confounding factors, including impaired leptin signalling and supra-pathological obesity, that need to be taken into consideration when interpreting findings (Barouch et al., 2003; Burke et al., 2017). Therefore, introducing a new experimental model to the toolkit of biomedical scientists, with a milder phenotype, will help us to further understand the underlying mechanisms of diabetic cardiomyopathy.

In this study we followed mice for 26 weeks from the induction of diabetes. As expected, given the results of our previous work, diabetic mice gained weight progressively over the duration of the study, primarily due to an increase in fat mass. Mice had a mild elevation in blood glucose and glycated haemoglobin, as well as a tendency for higher energy expenditure. A study in type 2 diabetes patients reported an elevation in 24 h energy expenditure (Bitz et al., 2004). Interestingly, studies have also shown that insulin signalling in the brain can influence whole body energy expenditure (Dodd and Tiganis, 2017; Loh et al., 2017). In relation to cardiac changes, the most prominent functional change in clinical diabetic cardiomyopathy is the presence of LV diastolic dysfunction (Loncarevic et al., 2016). In this study, diastolic dysfunction was evident in diabetic mice after 26 weeks of diabetes. This impairment developed in conjunction with myocardial fibrosis, a characteristic that has been reported in cardiac biopsies of patients with type 2 diabetes (Shimizu et al., 1993), as well as in several experimental models of diabetes (Wang et al., 2017; Tsai et al., 2018; Zhao et al., 2019).

An overproduction of ROS is a major driver of pathological remodelling in the diabetic heart (Kaludercic and Di Lisa, 2020). Clinical studies revealed elevated mitochondrial ROS production in cardiac biopsies of patients with type 2 diabetes (Anderson et al., 2009). Furthermore, elevated ROS has been reported in several experimental models of diabetes (Al-Rasheed et al., 2017; Li et al., 2019), including in the genetic models of diabetes, the *ob/ob* mouse and *db/db* mouse (Saraiva et al., 2007; Huynh et al., 2012). In the current study, diabetic mice displayed a significant increase in LV superoxide production, coupled with a tendency toward a decrease in LV hydrogen peroxide levels. Reduced superoxide dismutase activity, an antioxidant enzyme that catalyses the dismutation of superoxide into hydrogen peroxide, has been implicated in the setting of diabetic cardiomyopathy (Tang et al., 2018). A preclinical study has demonstrated that the expression of cardiac superoxide dismutase-2 was reduced in *db/db* mice (Waldman et al., 2018). Similarly, diabetic mice in the present study also exhibited a reduction in the mRNA expression superoxide dismutase-2. Our findings suggest that there is an impairment in the processing of ROS in the hearts of diabetic mice, possibly due to a reduction in amount or activity in superoxide dismutase. Superoxide generating enzyme known as NADPH-oxidase is also thought to contribute to oxidative stress in diabetic cardiomyopathy (Liang et al., 2018). However, in this study there was no difference in the mRNA or protein levels of the NADPH-oxidase subunit NOX-4.

The mitochondria are of particular importance in the diabetic heart as they are the major site for ROS production (Kaludercic and Di Lisa, 2020). Previous studies have demonstrated that mitochondrial dysfunction contributes toward the progression of diabetic cardiomyopathy (Marciniak et al., 2014; Wang et al., 2015; Ni et al., 2020). Adverse cardiac mitochondrial remodelling has been reported in 12-week-old *db/db* mice exhibiting decreased diastolic function and fractional shortening (Hu et al., 2019). In a similar study using STZ-mice, cardiac contractile dysfunction was associated with mitochondrial cristae fusion and reduced levels of adenosine triphosphate (Li et al., 2018). In diabetic rabbits, depolarised cardiac mitochondrial membrane potential and mitochondrial swelling was associated with diastolic dysfunction (Zhang et al., 2018). Our primary objective was to assess mitochondria structure and function in the myocardium of this alternative mouse model of diabetic cardiomyopathy. No differences were seen in the total number of mitochondria in the myocardium, however, diabetic mice exhibited a significant reduction in mitochondria size and mitochondrial disorganisation. Other studies in STZ-induced diabetic rodents have reported a reduction in both total number of cardiac mitochondria and relative size (Zhou et al., 2018; Tao et al., 2019). In the hearts of *db/db* mice, a decrease in mitochondria size and an increase in the number of mitochondria was observed; this was associated with decreased expression of mitochondria morphological regulator MFN but not DRP1 (Hu et al., 2019). Consistent with the *db/db* model, the protein expression of MFN2, but not DRP1, was also increased in diabetic mice (Hu et al., 2019). The downregulation of MFN2 may be partly the cause of smaller mitochondria size exhibited in diabetic mice in this model. These findings suggest that although cardiac mitochondrial structural changes are evident in the diabetic heart, morphological differences vary depending on the specific model and/or species. Increased availability of localised lipid droplets can impact mitochondrial dynamics in the diabetic heart by increasing the level of mitochondrial lipid uptake (Mazumder et al., 2004; Verma et al., 2017). In one study investigating cardiac tissue from diabetic mice, morphological changes in the mitochondria were parallelled with elevated mitochondrial fatty acid oxidation (Li et al., 2018). In our study, electron microscopy revealed an increased lipid-to-mitochondria ratio in diabetic mice.

Diabetes-induced changes in mitochondrial substrate utilisation can increase mitochondrial activity at the level of the respiratory complexes, leading to leakage of unpaired electrons and the formation of ROS (Alejandra Sánchez-Muñoz et al., 2018; Ding et al., 2019; Kaludercic and Di Lisa, 2020). A recent study demonstrated elevated protein expression of myocardial electron transport chain complexes (CI, CII, and CV) in diabetic mice (Wang et al., 2020). In this study there was an increase in complex-III and complex-V protein abundance, and a non-significant increase in complex-I and complex-IV. These findings indicate that a compensatory response to diabetes-induced excessive influx of reducing equivalents (NADH and FADH2) entering the mitochondrial complexes may be at play (Wu et al., 2017). This suggests a mismatch in energy expenditure (ATP consumption) and respiratory activity (reduced flow of electrons through the ETC) which may reflect the smaller mitochondria in the diabetic heart. Mitochondrial dysfunction is a known contributor to the pathological remodelling in diabetic cardiomyopathy (Ormazabal et al., 2018; Gollmer et al., 2020). Clinical studies have demonstrated a lower mitochondrial respiratory rate in the cardiac tissues of patients with diabetes (Croston et al., 2014; Montaigne et al., 2014). These findings also extend to experimental models of diabetic cardiomyopathy (Pham et al., 2014; Qaed et al., 2019). Consistent with previous studies, diabetic mice in this study exhibited similar reductions in LV mitochondrial oxygen consumption, particularly when measuring mitochondrial respiration by electron input through CII. Mitochondrial uncoupling proteins contribute to the protection of the myocardium by inducing mitochondrial respiration in response to a diabetes-induced reduction in the mitochondrial proton gradient (Dludla et al., 2018; Li et al., 2018). Studies have shown that an increase in mitochondrial uncoupling proteins is associated with decreased oxygen consumption (Hidaka et al., 1999; Pham et al., 2014; Fang et al., 2018). In this study, mRNA expression of mitochondrial UCP3 was significantly elevated with diabetes, suggesting that this may be a compensation mechanism in response to the diabetes-induced changes in substrate utilisation (Dludla et al., 2018; Li et al., 2018).

### Limitations and Future Considerations

We acknowledge that there were some limitations with our study which we discuss here. While only male mice were utilised in this study, we acknowledge the importance of investigating the pathological mechanisms of diabetic cardiomyopathy in female mice. We have previously demonstrated that STZ-induced female mice are more susceptible to diastolic dysfunction than male mice despite exhibiting lower degree of hyperglycaemia (Chandramouli et al., 2018). Further, female *db/db* mice exhibit at least as severe an adverse cardiac remodelling phenotype as male *db/db* mice (and indeed it may occur earlier) (Bowden et al., 2015). Another limitation of the present study was a lack of data reporting the respiratory exchange ratio (RER) which would have informed us about substrate utilisation and respiration. We attempted to measure this in our study, unfortunately, there was a failure of the data acquisition programme at the time of the Promethion metabolic cage recordings. The only data that we could retrieve for every mouse studied was the data for energy expenditure (Figure 3). Mitochondrial DNA copy number, which is a measure of the number of mitochondrial genomes per cell, can inform on mitochondrial function and has been associated with several disease states. Previous preclinical studies have shown that mitochondria DNA copy number is increased in diabetic heart (Boudina et al., 2007; Fang et al., 2018). After performing analysis on the mitochondria structure and function, the remaining heart tissue in this study was insufficient to carry out this analysis. This analysis however, is worthy of attention in future studies of mitochondrial function in the setting of diabetes. Previous studies using models of diabetic cardiomyopathy such as *db/db* mice and STZ-C57BL/6 mice have also demonstrated a decrease in ATP production with associated reduction in reducing equivalent (NAD + /NADPH ratio) (Boudina et al., 2007; Guan et al., 2015; Sun et al., 2019; Wang et al., 2020; Yao et al., 2021). Although our data demonstrated an increase protein abundance in ATP-synthase subunit (ATP-synthase-C5-subunit v) in diabetic mice (Figure 5), ATP levels and reducing equivalents were not measured as there was insufficient sample to carry out these analyses. There may be a maladaptive compensatory mechanism at play whereby defective ATP synthase is upregulated in the diabetic heart. Investigating these parameters in future studies may further elucidate the functional changes of cardiac mitochondria in this model of diabetic cardiomyopathy.

Despite these limitations and considerations, overall, our findings demonstrate the presence of mitochondrial dysfunction and excess ROS production in a mouse model that replicates several clinical features of diabetic cardiomyopathy. It is anticipated that this model which combines the low-dose STZ approach with high-fat diet, resulting in a milder phenotype compared to existing genetic models, can be used alongside existing models to study oxidative stress and mitochondrial function in the diabetic heart.

## Data Availability Statement

The raw data supporting the conclusions of this article will be made available by the authors, without undue reservation.

## Ethics Statement

The animal study was reviewed and approved by Alfred Medical Research and Education Precinct (AMREP) Animal Ethics Committee.

## Author Contributions

AP, MT, MJD, and RR performed conception and design of the research, drafted the manuscript, and edited and revised the manuscript. AP, MT, DP, MD, AW, DN, DD, SC, HK, CG, MC, MJD, and RR performed the experiments and approved final version of the manuscript. AP, MT, DP, MD, DN, DD, HK, MJD, and RR analysed the data. AP, MT, DP, DD, MJD, and RR interpreted results of the experiments. AP, MT, DD, HK, CG, MC, and RR prepared the figures. All authors contributed to the article and approved the submitted version.

## Conflict of Interest

The authors declare that the research was conducted in the absence of any commercial or financial relationships that could be construed as a potential conflict of interest.

## Publisher’s Note

All claims expressed in this article are solely those of the authors and do not necessarily represent those of their affiliated organizations, or those of the publisher, the editors and the reviewers. Any product that may be evaluated in this article, or claim that may be made by its manufacturer, is not guaranteed or endorsed by the publisher.

## Abbreviations

AMREP: Alfred Medical Research and Education Precinct
ANOVA: analysis of variance
AUC: area-under-the-curve
D: diabetic
H_2_O_2_: hydrogen peroxide
HbA1c: glycated haemoglobin
IPGTT: intraperitoneal glucose tolerance test
LV: left ventricular
ND: non-diabetic
ROS: reactive oxygen species
SEM: standard error of the mean
STZ: streptozotocin.
