# Supplementary material for: Characterisation of the Myocardial Mitochondria Structural and Functional Phenotype in a Murine Model of Diabetic Cardiomyopathy
Source: Front Physiol. 2021 Sep 1;12:672252. doi: 10.3389/fphys.2021.672252 (PMC8442993; doi:10.3389/fphys.2021.672252)
Supplement: Supplementary file 1 [file Presentation_1.PPTX]

## Slide 1
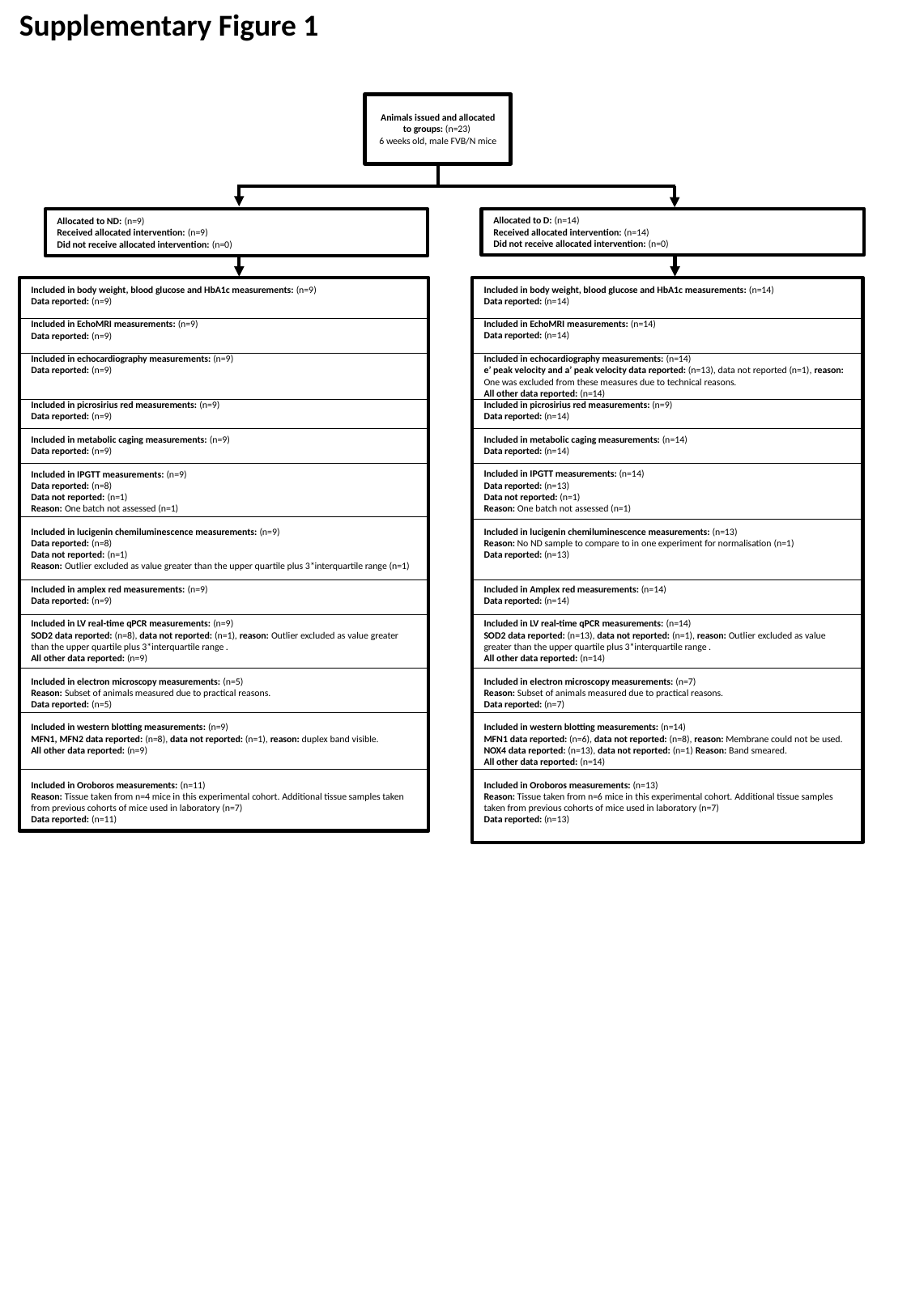

Supplementary Figure 1
Animals issued and allocated to groups: (n=23)
6 weeks old, male FVB/N mice
Allocated to D: (n=14)
Received allocated intervention: (n=14)
Did not receive allocated intervention: (n=0)
Allocated to ND: (n=9)
Received allocated intervention: (n=9)
Did not receive allocated intervention: (n=0)
Included in body weight, blood glucose and HbA1c measurements: (n=9)
Data reported: (n=9)
Included in EchoMRI measurements: (n=9)
Data reported: (n=9)
Included in echocardiography measurements: (n=9)
Data reported: (n=9)
Included in picrosirius red measurements: (n=9)
Data reported: (n=9)
Included in metabolic caging measurements: (n=9)
Data reported: (n=9)
Included in IPGTT measurements: (n=9)
Data reported: (n=8)
Data not reported: (n=1)
Reason: One batch not assessed (n=1)
Included in lucigenin chemiluminescence measurements: (n=9)
Data reported: (n=8)
Data not reported: (n=1)
Reason: Outlier excluded as value greater than the upper quartile plus 3*interquartile range (n=1)
Included in amplex red measurements: (n=9)
Data reported: (n=9)
Included in LV real-time qPCR measurements: (n=9)
SOD2 data reported: (n=8), data not reported: (n=1), reason: Outlier excluded as value greater than the upper quartile plus 3*interquartile range .
All other data reported: (n=9)
Included in electron microscopy measurements: (n=5)
Reason: Subset of animals measured due to practical reasons.
Data reported: (n=5)
Included in western blotting measurements: (n=9)
MFN1, MFN2 data reported: (n=8), data not reported: (n=1), reason: duplex band visible.
All other data reported: (n=9)
Included in Oroboros measurements: (n=11)
Reason: Tissue taken from n=4 mice in this experimental cohort. Additional tissue samples taken from previous cohorts of mice used in laboratory (n=7)
Data reported: (n=11)
Included in body weight, blood glucose and HbA1c measurements: (n=14)
Data reported: (n=14)
Included in EchoMRI measurements: (n=14)
Data reported: (n=14)
Included in echocardiography measurements: (n=14)
e’ peak velocity and a’ peak velocity data reported: (n=13), data not reported (n=1), reason: One was excluded from these measures due to technical reasons.
All other data reported: (n=14)
Included in picrosirius red measurements: (n=9)
Data reported: (n=14)
Included in metabolic caging measurements: (n=14)
Data reported: (n=14)
Included in IPGTT measurements: (n=14)
Data reported: (n=13)
Data not reported: (n=1)
Reason: One batch not assessed (n=1)
Included in lucigenin chemiluminescence measurements: (n=13)
Reason: No ND sample to compare to in one experiment for normalisation (n=1)
Data reported: (n=13)
Included in Amplex red measurements: (n=14)
Data reported: (n=14)
Included in LV real-time qPCR measurements: (n=14)
SOD2 data reported: (n=13), data not reported: (n=1), reason: Outlier excluded as value greater than the upper quartile plus 3*interquartile range .
All other data reported: (n=14)
Included in electron microscopy measurements: (n=7)
Reason: Subset of animals measured due to practical reasons.
Data reported: (n=7)
Included in western blotting measurements: (n=14)
MFN1 data reported: (n=6), data not reported: (n=8), reason: Membrane could not be used.
NOX4 data reported: (n=13), data not reported: (n=1) Reason: Band smeared.
All other data reported: (n=14)
Included in Oroboros measurements: (n=13)
Reason: Tissue taken from n=6 mice in this experimental cohort. Additional tissue samples taken from previous cohorts of mice used in laboratory (n=7)
Data reported: (n=13)

## Slide 2
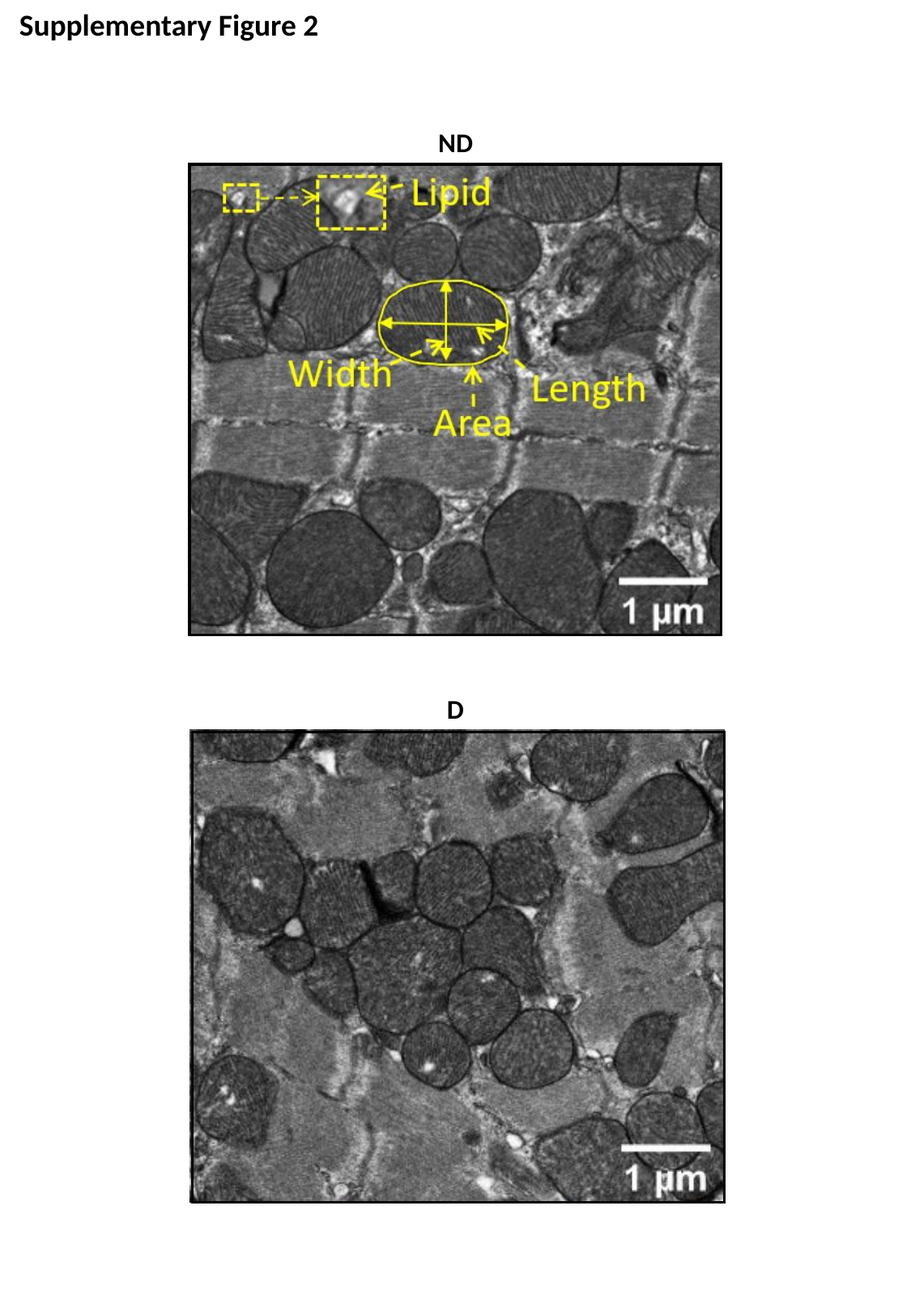

Supplementary Figure 2
ND
D
