# Supplementary material for: Characterisation of the Myocardial Mitochondria Structural and Functional Phenotype in a Murine Model of Diabetic Cardiomyopathy
Source: Front Physiol. 2021 Sep 1;12:672252. doi: 10.3389/fphys.2021.672252 (PMC8442993; doi:10.3389/fphys.2021.672252)
Supplement: Supplementary file 3 [file Data_Sheet_2.docx]

**Supplementary Table 1**

| **Gene name** | **Primer sequences (5'-3')** | |
| --- | --- | --- |
|  | **Forward** | **Reverse** |
| *18s* | TGTTCACCATGAGGCTGAGATC | TGGTTGCCTGGGAAAATCC |
| *CTGF* | TGACCCCTGCGACCCACA | TACACCGACCCACCGAAGACACAG |
| *NOX4* | CTGGAAGAACCCAAGTTCCA | GTTCATGAGCTGCCCACTGA |
| *UCP3* | CCAACATCACAAGAAATGC | TACAAACATCATCACGGTCC |
| *SOD2* | GCCCCCTGAGTTGTTGAATA | AGACAGGCAAGGCTCTACCA |

**Supplementary Table 2**

| **Organ weights, fat pad weights and B-mode/M-mode echocardiography analysis in control and diabetic mice (26 weeks of diabetes)** | | |
| --- | --- | --- |
|  |  |  |
|  | **Non-diabetic mice** | **Diabetic mice** |
| **Organ weights** | | |
| ***n*** | 9 | 14 |
| Tibial length (mm) | 17.9 ± 0.2 | 18.2 ± 0.1 |
| Heart weight (mg) | 134 ± 5 | 140 ± 3 |
| LV weight (mg) | 98 ± 4 | 101 ± 2 |
| Right ventricle weight (mg) | 21.5 ± 0.7 | 23.3 ± 0.8 |
| Atria weight (mg) | 11.1 ± 0.7 | 11.0 ± 0.7 |
| Kidney weight (left; mg) | 230 ± 11 | 218 ± 6 |
| Lungs weight (mg) | 161 ± 4 | 171 ± 6 |
| Liver weight (g) | 1.62 ± 0.05 | 2.03 ± 0.07* |
| Gastroc weight (mg) | 163 ± 3 | 165 ± 3 |
| Spleen weight (mg) | 103 ± 5 | 123 ± 7* |
| Pancreas weight (mg) | 229 ± 11 | 205 ± 7 |
| **Fat pad weights** | | |
| Peri-cardial fat weight (mg) | 19.5 ± 3.3 | 29.5 ± 4.1 |
| Epididymal fat weight (g) | 1.18 ± 0.10 | 1.22 ± 0.09 |
| Peri-renal fat weight (g) | 0.68 ± 0.06 | 1.01 ± 0.07* |
| Inguinal fat weight (g) | 0.62 ± 0.05 | 1.31 ± 0.14* |
| **M-mode echocardiography** | | |
| ***n*** | 9 | 14 |
| Heart rate (bpm) | 382 ± 12 | 364 ± 6 |
| AWd (mm) | 0.60 ± 0.01 | 0.64 ± 0.01 |
| PWd (mm) | 0.62 ± 0.02 | 0.67 ± 0.02 |
| LVEDD (mm) | 4.33 ± 0.13 | 4.36 ± 0.07 |
| LVESD (mm) | 3.09 ± 0.15 | 3.07 ± 0.06 |
| Fractional shortening (%) | 29.0 ± 1.6 | 29.5 ± 0.8 |
| **B-mode echocardiography** | | |
| ***n*** | 8 | 14 |
| Systolic area (mm^2^) | 15.8 ± 0.4 | 15.3 ± 0.5 |
| Diastolic area (mm^2^) | 22.5 ± 0.6 | 22.1 ± 0.3 |
| Systolic volume (μL) | 32.9 ± 1.6 | 31.6 ± 1.5 |
| Diastolic volume (μL) | 59.6 ± 2.7 | 59.1 ± 1.1 |
| Stroke volume (μL) | 26.7 ± 1.7 | 27.5 ± 1.4 |
| Ejection Fraction (%) | 44.6 ± 2.0 | 46.6 ± 2.3 |
| Cardiac Output (mL/min) | 11.0 ± 0.9 | 10.5 ± 0.6 |
| **Data are presented as mean ± SEM and analysed by unpaired t-test. *P<0.05 vs non-diabetic mice. Awd, anterior wall diastolic thickness; LVEDD, LV end diastolic dimension; PWd, posterior wall diastolic thickness; LVESD, LV end systolic dimension** | | |
